# 72 Distinguishing features of MIS-C to other paediatric febrile diseases in the acute setting

**DOI:** 10.1093/rheumatology/keac496.068

**Published:** 2022-10-07

**Authors:** Claire Butters, Timothy Spracklen, Raphaella Stander, Jonathan Day, Heidi Facey-Thomas, Liesl Zuhlke, Christiaan Scott, Kate Webb

**Affiliations:** 1 Department of Paediatrics and Child Health, University of Cape Town, Cape Town, South Africa; 2 Division of Immunology, Institute of Infectious Disease and Molecular Medicine, University of Cape Town, Cape Town, South Africa; 3 Cape Heart Institute, University of Cape Town, Cape Town, South Africa; 4 South African Medical Research Council, Cape Town, South Africa; 5 Clinical Research Centre, University of Cape Town, South Africa; 6 The Francis Crick Institute, Crick African Network, London, UK

## Abstract

**Background:**

Multisystem inflammatory syndrome in children (MIS-C) presents with fever, shock, rash, abdominal pain and raised inflammatory markers, as well as common features of inflammatory childhood illnesses. In the acute setting, especially in countries where infectious diseases are common differential diagnoses, it is challenging to diagnose MIS-C. Therefore, data differentiating MIS-C from other inflammatory and/or febrile diseases at presentation is needed.

**Methods:**

Prospective data was collected from children admitted to the Red Cross War Memorial Children’s Hospital in Cape Town, South Africa from May 2020 to end November 2021 where MIS-C was part of their differential diagnoses. Clinical features on the day of admission were compared between children with confirmed MIS-C (MIS-C+) and those with alternate diagnoses (MIS-C-).

**Results:**

In this time period, 60 children were MIS-C+ and 34 were MIS-C-. There was no significant difference in age (*p* = 0.321), sex (*p* = 0.525), ethnicity (*p* = 0.279), or in the frequency of comorbidities (*p* = 0.151) between the two groups. The presence of conjunctivitis (OR = 8.12), rash (OR = 8.67), tachycardia (OR = 2.8) and oral mucositis (OR = 3.75) was associated with MIS-C+ while abdominal pain and hypotension were not. MIS-C+ had statistically higher median C-reactive protein (CRP), pro-brain natriuretic protein (pro-BNP) and ferritin, and lower median lymphocyte count, platelet count and sodium levels than MIS-C-. Ferritin discriminated MIS-C+ well (AUC = 0.86) with a 94% sensitivity and 60% specificity at a cut off of > 195ng/l. Sodium had an AUC of 0.72, with a 70% sensitivity and 71% specificity at a cut off of < 132.5 mmol/l. CRP did not distinguish MIS-C well (AUC = 0.52) and although they had good AUC, platelet count and pro-BNP had cut off values in the normal range decreasing clinical utility.

**Conclusion:**

We provide evidence for the use of accessible clinical and laboratory variables for the diagnosis of MIS-C in diverse settings.

**Implications:**

These data will aid clinicians to do a rapid diagnosis (and ultimately treat earlier) of patients with MIS-C in the acute setting, especially those in under-resourced settings.

**72 Table 4:** ROC analysis of laboratory tests which were different between MIS-C- and MIS-C+

Test	AUC (95%CI)	p	Potential cut-off value	Sensitivity	Specificity	Youden ratio
Ferritin	**0.86 (0.70, 1)**	**0.017**	*>195	94%	60%	**54%**
7-84 ng/L			^**^>316	72%	80%	**52%**
Pro-BNP	**0.80 (0.62, 0.98)**	**0.044**	*>239	83%	40%	23%
<450 ng/L			^**^>378	78%	80%	**57%**
Platelet count	**0.75 (0.59, 0.90)**	**0.006**	*<285	81%	71%	**52%**
180-440 x10^9^/L						
Sodium	**0.726 (0.56, 0.88)**	**0.014**	*<134.5	87%	35.7%	22%
136-145 mmol/L			^**^<132.5	70%	71%	41%
CRP	0.54 (0.62, 0.98)	0.766	*>77	100%	40%	40%
<10 mg/L			^**^>161.5	50%	60%	10%

2 potentials cut off values are given.

* the value which gives the best specificity with a sensitivity >80% and.

** the value that achieves the best sensitivity with a specificity of at least 60%.

